# Endothelial Dysfunction in Systemic Lupus Erythematosus and Systemic Sclerosis: A Common Trigger for Different Microvascular Diseases

**DOI:** 10.3389/fmed.2022.849086

**Published:** 2022-04-08

**Authors:** Liala Moschetti, Silvia Piantoni, Enrico Vizzardi, Edoardo Sciatti, Mauro Riccardi, Franco Franceschini, Ilaria Cavazzana

**Affiliations:** ^1^Rheumatology and Clinical immunology Unit, ASST Spedali Civili of Brescia, Department of Clinical and Experimental Sciences, University of Brescia, Brescia, Italy; ^2^Cardiology Unit, ASST Spedali Civili of Brescia, Department of Medical and Surgical Specialties, Radiological Sciences and Public Health, University of Brescia, Brescia, Italy; ^3^Cardiology Unit 1, ASST Papa Giovanni XXIII, Bergamo, Italy

**Keywords:** endothelial dysfunction, systemic lupus erythematosus, systemic sclerosis, microvascular disease, techniques of assessment

## Abstract

This review describes the complex interplay between inflammation, vasculopathy and fibrosis that involve the heart and peripheral small vessels, leading to endothelial stiffness, vascular damage, and early aging in patients with systemic lupus erythematosus and systemic sclerosis, which represents two different models of vascular dysfunction among systemic autoimmune diseases. In fact, despite the fact that diagnostic methods and therapies have been significantly improved in the last years, affected patients show an excess of cardiovascular mortality if compared with the general population. In addition, we provide a complete overview on the new techniques which are used for the evaluation of endothelial dysfunction in a preclinical phase, which could represent a new approach in the assessment of cardiovascular risk in these patients.

## Introduction

Systemic autoimmune diseases are disorders characterized by humoral and cell-mediated immune responses against various self-antigens. A higher cardiovascular (CV) morbidity and mortality rates were described in affected patients (1). Persistent low-grade inflammation in the vascular wall is considered the crucial trigger for CV events through endothelial dysfunction (ED) and proliferation of vascular smooth muscle cells, with subsequent vascular remodeling (2). Furthermore, the infiltration of different immune cells promotes a *milieu* of molecules that contributes to the perpetuation of inflammation itself. ED is currently considered the main mechanism explaining the microangiopathy in different clinical autoimmune conditions. An insufficient endothelium-dependent vasodilation in reply to vasoactive stimuli, principally due to the failing production of nitric oxide (NO) and/or an impaired NO function, defines ED. ED has been detected in different types of arterial vessels, and actually it is considered a systemic process (3, 4). Among systemic autoimmune diseases, ED has been extensively studied in systemic lupus erythematosus (SLE) and systemic sclerosis (SSc), which represent two different models of ED dysfunction. In SLE patients, ED is the main actor of vascular aging and pre-clinical atherosclerosis during the course of the disease, contributing to the early onset of CV disease (CVD) and CV mortality. On the other hand, in SSc, ED and microangiopathy are key factors sustaining the development of the disease itself. The aim of this review is to analyze the factors which has a role in the pathophysiology of ED in SLE and SSc and to explore the new techniques which could be used in its evaluation in a pre-clinical phase. In fact, traditional Framingham risk factors do not fully explain the increased CV risk in rheumatic diseases (5) and, although CV risk assessment should be part of routine assessment in patients, no disease-specific models are currently available for this purpose (6, 7). Recently, the European Alliance of Associations for Rheumatology (EULAR) published some recommendations for CV risk management in these patients, suggesting the need of a precocious diagnosis without the endorsement of the use of any particular assessment tool (8).

## Systemic Lupus Erythematosus and Endothelial Dysfunction

Systemic lupus erythematosus is a chronic systemic autoimmune disease characterized by a dysregulation of immune system, leading to autoantibody production, tissue inflammation, and organ damage. Since approximately 40 years, SLE is known to display a raised mortality, due to premature CVD (4). Compared to the general population, the prevalence of CVD is known to be at least double in SLE patients (9, 10), especially in young premenopausal women (11). Accelerated atherosclerosis, estimated to develop or progress in 10% of SLE patients each year (12) and that is globally sixfold more frequent in SLE compared with the general population (13), is associated to this premature CVD. Although a high cardiometabolic risk has been described in SLE (14), CVD in SLE displays atypical features, such as presentation in young women and a lack of a clear protective effect by statins (15, 16). Early CVD in SLE is known to be associated with ED and stiffness of vascular tree, that lead to atherosclerosis and clot formation, involving different pathogenetic mechanisms (17).

### Pathogenesis of Endothelial Dysfunction in Systemic Lupus Erythematosus

Several mechanisms have been proposed to explain ED and atherosclerosis in SLE (18, 19), resulting in a clear predominance of injury stimuli versus protection factors on the layer of endothelial cells (ECs).

#### Oxidative Stress

Mitochondrial dysfunction and abnormal telomere/telomerase balance lead to a persistent oxidative stress in SLE, mainly involving circulating leukocytes and ECs (20). The oxidative process induces cell adhesion molecules (CAMs) expression (21), with consequent higher leucocyte-endothelial cell interactions and leucocytes’ transmigration to sites of inflammation (22). In addition, a significant association between higher anti-double stranded-DNA (anti-dsDNA) antibodies levels and higher levels of oxidative products was reported (23, 24). The excessive production of reactive oxygen and nitrogen species (ROS and RNS) leads to modifications of different cellular molecules, such as proteins, lipids, deoxyribonucleic acid (DNA) or ribonucleic acid (RNA), generating neo-antigens with a consequent production of autoantibodies, and uncontrolled lymphocytes’ activation (23, 25). In SLE, three main targets of oxidative stress have been identified: oxidized lipids, oxidized low-density lipoprotein (LDL) and proinflammatory high-density lipoprotein (HDL), all playing a crucial role in pathogenesis of SLE-related ED and atherosclerosis (26, 27).

#### Cytokine Cascade

Proinflammatory cytokines play a direct role in accelerating SLE atherosclerosis. In particular, all three classes of interferons (IFNs), namely IFN-I (IFN-α, IFN-β, IFN-δ, IFN-ε, IFN-κ, IFN-τ, IFN-ω, and IFN-ζ), IFN-II (IFN-γ), IFN-III (IFN-λ1, IFN-λ2, and IFN-λ3), participated in the process of atherosclerosis (19). IFN-α and IFN-γ promote lipoproteins’ oxidation (28, 29) and ED by accelerating ECs apoptosis and damaging endothelial progenitor cells (EPCs) (28, 30), one of the vascular repair mechanisms. On the other hand, IFN-γ increases vascular smooth muscle cells’ (VSMC) proliferation and migration (31), VSMC and macrophages apoptosis in atherosclerotic plaques, inducing plaque instability (32). The long-term activation of IFN-I system induces the expression of different chemokine pathways that recruit leukocytes into inflammatory sites promoting the dysfunction of ECs and EPCs (19).

#### Neutrophil Extracellular Traps

Neutrophil Extracellular Traps (NETs), a unique type of neutrophils communication characterized by the extrusion of chromatin and other molecules, are considered a key factor in SLE atherosclerosis (33). NETs can enhance vascular leakage, endothelial-to-mesenchymal transition (34) and ECs death (35). Moreover, NETs enhance oxidation processes (36), secretion of IFN-α (37), interleukin (IL)-1β (38), and activate coagulation cascade (39).

#### B Cells and Autoantibodies

Many autoantibodies can affect endothelial function, by promoting pathogenic molecules and inhibiting potential protective factors (40). Antiphospholipid antibodies (aPL), that are anticardiolipin antibodies (aCL) and anti-β2-glycoprotein I antibodies (anti-β2GPI), can contribute to accelerated atherosclerosis by inducing a proinflammatory endothelial phenotype through a direct interaction with ECs (41). Different authors described ECs activation by aPL via EC-derived extracellular vesicles through a toll like receptor (TLR) 4 and 7-dependent pathway, resulting in paracrine stimulation of neighboring unstimulated ECs (42–44). In addition, aPL can upregulate the tissue factor expression on ECs and monocytes, and promote endothelial leukocyte adhesion and pro-inflammatory cytokine secretion (41). Finally, aPL are considered an independent predictor of atherosclerotic plaque progression in SLE (45). Other autoantibodies have been described as contributors of accelerated atherosclerosis in SLE: anti-HDL-IgG that induce LDL to enter the ECs; anti-apolipoprotein A1 (ApoA1)-IgG that activating the transcriptional nuclear factor kappaB (NF-kB) favor the expression of inflammatory factors at endothelial level (46); anti-FXa-IgG can inhibit FX enzyme (47), modifying the of hemostasis/thrombosis equilibrium and promoting ED (48). Moreover, anti-C1q antibodies play a role in atherosclerosis by reducing C1q’s level and lowering their protective effects on endothelium (49, 50).

#### T Cell Subpopulations

In general, the subset of CD28- (CD28null) T cells is char- acterized by pro-inflammatory properties and plays an active role in destabilization of the plaque itself, increasing endothelial oxidative markers, and arterial stiffness (51). In humans, high levels of CD4+CD28null T cells, responsible of an aberrant T-B lymphocytes’ interaction, have been described during instable angina, and could be involved in the atherosclerotic plaque instability (52). The prevalence of these cells is increased in systemic autoimmune diseases because of the repeated antigenic stimulation that induces a downregulation of CD28 from the lymphocytes’ membrane (53). The so-called angiogenic T cells (Tang) are characterized by the expression of CD3, the platelet-endothelial cell adhesion molecule-1 (CD31) and the receptor for stromal cell factor-1 CXCR4 (54). Due to their ability to enhance endothelial repair function (55) and promote new vessel formation (54), Tang could be used as a novel putative biological marker for CVD. A higher number of circulating Tang may be involved in ED among several autoimmune diseases, such as rheumatoid arthritis (RA), SLE and SSc, as a consequence of endothelial damage or an inefficient angiogenesis (56–58). Accordingly, in a recent study of our group, we demonstrated that the nail video-capillaroscopy (NVC) alterations in a cohort of patients with SLE and without traditional CV risk factors were associated with ED and with the increase of circulating Tang (59). A subtype of Tang called “aging” Tang (CD28null-Tang) seems to be not protective but cytotoxic, due to their ability to secrete inflammatory mediators and release cytolytic molecules from intracellular particles, inducing EC damage and accelerated atherosclerosis in most SLE patients (60). Moreover, CD28null-Tang increased in SLE patients with traditional CV risk factors and active disease (60). In our recent experience, we observed that the rate of circulating pro-angiogenic Tang decreased very early in disease course, with an increase of the rate of the “aging” CD28null subset. Our preliminary data suggest that Tang might exert their effects on the endothelium via the pro-angiogenic mediators IL-8 and metalloproteinase-9 (61). Another T lymphocyte subtype, regulatory T cells (Treg), are believed to play a protective role in autoimmune diseases. Anyway, atherosclerosis’s severity does not seem to be strictly related to their numbers, but rather to their dysfunction (62, 63). In SLE, Treg cells are significantly reduced in both, number and function (64). In human studies and mouse models, Treg have been associated with a protective role in atherosclerosis (65) and their decrease is significantly associated with acute coronary events (18). Recently, the invariant natural killer T cells showed an anti-atherosclerotic phenotype in SLE patients and can induces macrophages to polarize into anti-inflammatory and anti-atherosclerotic M2 phenotype (66).

#### Endothelial Progenitor Cells

Endothelial progenitor cells are a group of bone marrow-derived cells, acting in vascular homeostasis control and endothelial repair (67). Some authors reported a reduced number of EPCs in patients with CV risk factors (68) and CVD (69). Therefore, EPCs could be considered a new marker of CV risk, especially in SLE patients in which traditional CV prediction models fail to estimate the risk of clinical CVD. Physiologically, after endothelial injury, vascular repair occurs by accelerating the replacement of ECs: a process that involves proliferation and migration of adjacent ECs and resident EPCs and recruitment of new EPCs. Although data in SLE are controversial, EPCs are reduced in number and are functionally impaired (19). This impairment seems to be the result of the balance between risk factors (including IFN-I) and protective factors (including Tang cells). In particular, IFN-I accelerates SLE atherosclerosis, by interfering with EPCs (19), as suggested by studies in adult- or childhood-onset SLE (67, 70). The results among studies are difficult to be compared because EPCs could be identified using different and not yet standardized methods, such as flow cytometry or through different cell isolation techniques (67). Type I IFN, overexpressed during a SLE flare and involved in SLE pathogenesis, was described as a contributor of EPCs dysfunction in the disease (67). Furthermore, some data demonstrated that recombinant IFN-α displays a toxic effect on CD133/CD34 + cells (e.g., putative EPCs) in culture. The use of monoclonal antibody blocking IFN pathways in SLE leads to a normalization of EPCs function (71).

### Cardiovascular Disease Risk Assessment in Systemic Lupus Erythematosus

Systemic lupus erythematosus represents a good example of autoimmune disease associated to an inflammatory-related early atherosclerosis. It is widely known that SLE patients have a significant risk of CVD, presenting a higher rate of atherosclerotic large arterial vessels, as well as in RA and diabetes mellitus (72). Furthermore, as compared to the general population, SLE patients have a twofold increased rate of ischemic myocardial infarction (73, 74). The presence of lupus nephritis and aPL represents further risk factors for CVD in SLE (75). According with guidelines (8), the assessment of traditional but also the disease-related risk factors is recommended in SLE patients. A modified version of the Framingham risk score that used 2 as multiplicative factor was showed to increase the sensitivity in identifying patients with an increased risk of coronary artery disease (76). It became necessary to develop a SLE-specific CV risk score that combines traditional CV risk factors and SLE-specific variables: only disease activity score, C3 level, and lupus anticoagulant titer were predictive of CV outcomes (77). Petri et al., determined that patients with higher SLE disease activity index (SLEDAI) score had their 10-year risk underestimated by as much as a factor of 10 (78). Inaccurate CVD risk assessment is evident especially in young SLE patients, that are not likely to experience adverse CV events within 10 years: for these patients a more complex and multidisciplinary risk assessment appears of utmost relevance (78). In SLE patients, levels of blood pressure lower than 130/80 mmHg are recommended because are associated with lower incidence of CV manifestations (8). For the other risk factors, treatment suggestions should follow recommendations that are used among general population. The impact of most used immunosuppressant agents in SLE on accelerated atherosclerosis has been understudied and, actually, any drugs could be recommended with the purpose of lowering CV risk (77). The maintenance of a low disease activity was demonstrated to be a good strategy to reduce CV risk among these patients, such as the limitation of the use of glucocorticoids to the lowest effective dose considering their well-known deleterious cardiometabolic effects (8, 79). Selective B cell activating factor (BAFF) inhibition, belimumab, seems to display a double effect in animal models: in low-lipid conditions, BAFF inhibition is predictably athero-protective, but in high lipid environments it is atherogenic, due to a counter function in macrophages (80). Hydroxychloroquine shows multiple protective effects (77), reducing IFN-α production, aortic stiffness, correcting lipoprotein profile, improving glycemic control, as well as reducing the risk of all thrombo-vascular events in SLE patients. Finally, mycophenolate treatment seems to improve HDL function in SLE patients, and reduces atherosclerosis mouse models, limiting the recruitment of CD4 + T cells to atherosclerotic lesions (81). Preventive strategies, such as the introduction of low-dose aspirin, is based on individual CV risk profile which should include the assessment of aPL which are more frequent in SLE than in general population (8).

## Systemic Sclerosis and Endothelial Dysfunction

Systemic sclerosis is a rare, acquired, systemic disease of unknown origin and uncertain pathophysiology characterized by multi organ involvement. Vascular alterations, extensive fibrosis and specific autoantibodies are the principal actors of its pathogenesis (82). While in SLE ED and accelerated atherosclerosis are a consequence of the chronic and sustained inflammation (83), in SSc microvascular dysfunction is one of the hallmarks of the disease along with immune dysregulation and widespread fibrosis, and represents a primary pathogenetic process (84). Indeed, vasculopathy is of fundamental importance in SSc, from the very early onset of the disease, manifesting with Raynaud’s phenomenon that usually precede the other disease manifestations, through the late clinical complications whose prototype is the pulmonary arterial hypertension (PAH). These widespread vascular abnormalities can also present as ischemic digital ulcers (DU), mucocutaneous telangiectasias, gastric antral vascular ectasia and scleroderma renal crisis (85).

### Pathogenesis of Microangiopathy in Systemic Sclerosis

#### Oxidative Stress

Repetitive ischemia and reperfusion processes causes oxidative stress with subsequent tissue damage in SSc, mediated by proinflammatory cytokines and activated leukocytes. These activated leukocytes also show increased expression of inducible nitric oxide synthase (iNOS), leading to the production of a huge amount of NO that reacts with oxygen in the re-perfused blood to form ROS. This causes a direct endothelial injury that leads to vasoconstriction and conversion to a procoagulant phenotype (86).

#### Endotheliitis

The dysregulation of EC within the vascular wall has a major role in the above-mentioned fibroproliferative vasculopathy (87). This contribute to the unbalanced production of vasoactive mediators resulting in vasoconstriction (88, 89). The alterations of mediators involved in this process were described as both quantitative and qualitative. A particular mention has to be done with regards to the alterations of the vascular endothelial growth factor (VEGF). In fact, despite the fact that higher circulating levels of this vasodilator agent were described in SSc patients in comparison with healthy controls, anti-angiogenic VEGF isoform was strongly expressed in the skin of SSc patients (90). In addition, the increased expression of adhesion molecules by damaged endothelial surface promotes leukocyte trans-endothelial migration, activation, and accumulation (91, 92). ECs transdifferentiate into myofibroblasts gaining mesenchymal cell markers (93, 94). These events culminate in the intima-media proliferation and vessel occlusion leading to tissue hypoxia, which further promotes cell injury and fibroblasts activation (87). Viral infections, coagulation cascade activation, complement system impairment and antibodies against ECs have been proposed as the initial trigger in SSc pathogenesis (95, 96). Some viral infections have been linked to activation/injury of ECs through a mechanism of molecular mimicry. For instance, human cytomegalovirus infection induces antibodies that recognize an amino acid sequence on a viral protein, which is homologous to a surface molecule highly expressed on ECs, inducing apoptosis of ECs (97). Some studies have found a correlation between the parvovirus B19 DNA expression levels and the severity of ED in SSc (98, 99). Recently, new evidence focuses on whether SARS-CoV-2 infection triggers autoimmunity and may have a role in SSc pathogenesis. Indeed, exploration of the SARS-CoV-2-related endotheliitis might provide further important information in the understanding of the early SSc pathogenesis (100).

#### Complement System

The complement system role in the pathogenesis of SSc vasculopathy has not been exhaustively studied. Its classical functions such as opsonization, recruitment of inflammatory cells, influence of coagulation cascade and angiogenesis are primary for ECs integrity. In normal conditions, complement attack is tightly regulated by regulatory proteins, ensuring protection of EC layer. A reduced expression of these regulators has been shown in SSc skin, potentially leading to endothelium-bound membrane attack complex of complement deposition that could cause EC apoptosis (101).

#### Autoantibodies

The anti-endothelial cell antibodies (AECAs) can be found in almost 50% of SSc patients and can react with various cell surface antigens on ECs leading them to apoptosis (102) through the antibody-dependent cell-mediated cytotoxicity mechanism (103–105). An association between circulating antibodies and vascular manifestations has been described for antibodies against cell surface receptors such as angiotensin II type 1 receptor and endothelin-1 type A receptor (106). Among other antibodies possibly associated with vasculopathy in SSc, aPL should be considered. Their frequency in SSc is highly heterogeneous and ranges from 0 to 57% (107). Sobanski et al., carried out a meta-analysis, revealing an overall pooled prevalence of 14% (108). ACL and anti-β2-GPI antibodies can contribute to accelerated atherosclerosis by interacting with ECs and inducing a proinflammatory endothelial phenotype (41). Some studies reported an association between aPL positivity and PAH and DU (109–113), while others did not (108, 114, 115). Lastly, considering the strong clinical associations of SSc specific antibodies (anticentromere, anti-topoisomerase 1, anti-RNA polymerase III and anti-Th/To antibodies) and their role as prognostic biomarkers, a potential pathogenicity of these antibodies was suggested. Raschi et al., demonstrated that SSc specific antibodies bound to their antigens to form immune complexes (ICs) elicit pro-inflammatory and pro-fibrotic effects on healthy ECs (95). They stated that immune complexes composed with SSc specific antibodies might contribute to scleroderma pathogenesis through a direct interaction with TLRs. Endothelial incubation with SSc-ICs modulates several molecules (endothelin-1, IL-8, inter-CAM-1, IL-6, and transforming growth factor β1) involved in the three cardinal scleroderma pathophysiologic processes (95).

#### T Cell Subpopulations

As previously outlined, Tang are required for endothelial progenitor colony formation, promote new vessel formation by secreting angiogenic factors such as VEGF and adhere to ECs. Tang can interact directly with the CD31 expressed by ECs via endothelial-T-cell CD31-CD31 homophilic interactions. In addition, given that these cells also express the cytotoxins granzyme B and perforin, they also have cytotoxic potential. Zhang et al., reported that these cells secrete large amounts of proinflammatory cytokines, such as tumor necrosis factor alpha, IL-6 and IFN-γ, confirming their proinflammatory features (116). Interactions these Tang related cytokines may contribute to ED by exacerbating oxidative stress and reducing phosphorylation of endothelial NOS (117). Their frequency is increased in individuals with traditional CV risk factors further supporting their role in regulating ED (60). It was found that circulating Tang were significantly increased in SSc patients with DU compared either with SSc patients without DU or with healthy controls. In addition, in SSc patients, Tang levels correlate with NVC patterns: higher levels were observed in patients presenting late NVC pattern more frequently than in those with early/active NVC patterns (58). In another study, the absolute number of Tang was higher in SSc patients compared to healthy controls, especially in SSc patients with PAH (118). Taken together, these findings demonstrated that Tang are expanded in SSc patients displaying severe peripheral vascular complications suggesting that circulating Tang increase as a reaction to ischemia and might represent a novel biomarker closely reflecting the severity of SSc-related peripheral vasculopathy.

#### Endothelial Progenitor Cells

The scleroderma impairment of neovascularization could be associated to both angiogenesis and vasculogenesis failure. Besides insufficient angiogenesis, the contribution of defective vasculogenesis to SSc vasculopathy has been extensively studied (119). As mentioned above, EPCs are defined as circulating primitive cells that contribute to postnatal vasculogenesis (120) and, in SSc patients, circulating EPCs were shown to be reduced in comparison with healthy controls (121). In addition to quantitative alterations, an impaired potential of SSc-derived EPCs to differentiate into mature ECs was reported in terms of functional properties of EPCs (122). It was suggested that EPC precursors were functionally altered before their release into the bloodstream because of a dysregulated microenvironment within the bone marrow (reduced microvascular density and increased fibrosis) (123, 124). In addition, the hypoxic condition of the affected tissues of SSc patients are known to stimulate the differentiation of monocytic EPCs, one EPCs subset (125), through activation of hypoxia-inducible factor (HIF)-1α (126). These local stimuli promote the accumulation of functionally altered monocytic EPCs into the affected lesions of SSc and, since monocytic EPCs are capable of differentiating into cells that produce extracellular matrix proteins (127, 128), they might participate in the fibrotic process in the affected organs (128, 129).

### Cardiovascular Disease Risk Assessment in Systemic Sclerosis

SSc patients are at a higher risk of atherosclerosis, albeit, its pattern appears to be less aggressive compared with other rheumatic diseases (130). The alteration of microvasculature is a main feature of SSc and a central cause of complications, but also a macrovascular dysfunction was described (131). In fact, a high incidence of coronary artery disease among SSc patients was reported (132). Among all the connective tissue diseases, SSc is currently associated with the highest mortality rate, with an estimated 10-year survival of 66–82% (133). Due to the recent improvements in the treatment, SSc patients are dying less from SSc-related complications and more from non-SSc related causes, which now account for about 50% of all SSc deaths (133). CVD contributes significantly to SSc mortality burden, accounting for 20–30% of all SSc deaths. For this reason, an accurate understanding of CV risk is crucial in order to improve the overall outcomes of SSc patients (86). However, recommendations for cardiac assessment, CVD risk stratification and prevention strategies in this particular population are currently lacking (134). All patients with SSc should undergo a full evaluation for conventional CV risk factors, even if, compared to general population, the prevalence of traditional CV risk factors in SSc do not seem to differ significantly (135). Standard therapies have to be considered in this context. Early treatment with calcium channel blockers (CCBs), angiotensin-converting enzyme inhibitors, and endothelin receptor antagonists (ERAs), were demonstrated to be efficacious on myocardial perfusion and contractility, as they improve cardiac microcirculation (136). Vasodilator agents such as phosphodiesterase-5 inhibitors, reducing circulating cytokines and chemokines and suppressing oxidative stress, can improve endothelial function in the patients (137). According with the last published recommendations (8), the management of blood pressure and of hyperlipidemia in these patients should follow the rules used in general population, without specific indications about the use of low-dose aspirin for the prophylaxis.

## Endothelial Dysfunction Assessment

The first demonstration of ED in atherosclerotic patients was done using intracoronary infusion of acetylcholine by Ludmer and colleagues in the nineteenth century, heralding an important shift in the paradigm of human atherosclerosis regarded as a purely structural disease (138). Later, several and less invasive techniques to detect changes in the morphology and function of the microcirculation at subclinical level have been developed. The forearm circulation but also the retinal capillary bed was considered as a surrogate for coronary arteries (138). These techniques were mostly applied to primary CVD, except for NVC which is applied in the routinely SSc evaluation In this review we focused the attention on techniques evaluating peripheral circulation.

### Nailfold Video-Capillaroscopy

Nailfold video-capillaroscopy is a non-invasive and reproducible imaging study of capillary circulation which is easily accessible in daily routine. It is a well-documented and established tool for the evaluation of peripheral microcirculation in SSc and it has been incorporated in the last international SSc classification criteria (139, 140). The specific alterations which are recognized in SSc form a characteristic morphological pattern known as “scleroderma pattern” (141). The “early” pattern is characterized by few enlarged/giant capillaries, few hemorrhages and relatively well-preserved capillary distribution with no evident loss; the “active” pattern is defined by frequent giant capillaries and hemorrhages and by mild disorganization of the architecture with moderate loss of capillaries; the “late” pattern is characterized by the disorganization of the normal capillary array and the presence of scarce capillaries which show irregular enlargement with ramified/bushy structure (139). Over the last years, the implications of NVC have expanded beyond the diagnostic evaluation of Raynaud’s phenomenon to the point that NVC patterns are considered as potential surrogate markers of disease severity and of disease progression (142). Morphological vascular patterns are correlated to the severity of SSc as they seem to reflect the different phases of the disease. The early pattern characterizes the incipient vascular changes and the active/late patterns represents the extensive capillary damage characterizing the fibrotic phase of SSc (143). Indeed, several studies have investigated the association between NVC and SSc manifestations finding some associations of NVC alterations to PAH (144–146) and to telangiectasias (147, 148). However, these data were not confirmed throughout the studies on the topic (149–151). In view of ED and CVD risk in SSc patients, NVC patterns have been associated with arterial stiffness and CVD risk scores supporting a link between micro and macrovascular damage in this disease (152, 153). Limited data exist on the use of NVC in SLE. Many different capillary forms and patterns and a variable prevalence of capillary abnormalities has been reported. In morphometric studies longer capillaries have been described as characteristics of SLE, while in the presence of an associated antiphospholipid syndrome the typical NVC findings are called “comb-like” hemorrhages and consists in multiple hemorrhages from normal shaped capillaries (154, 155). Non-specific morphological alterations, can be found in approximately 75% of SLE patients and relevant capillaroscopic changes correlate with disease activity and with the presence of anti-U1RNP antibodies and aCL (154). However, reported data on association between these findings and disease-related organ involvement are conflicting (156). In addition to morphological and structural evaluation of capillary bed, a dynamic method for studying skin capillaries has been applied to NVC, based on the principle of reactive hyperemia after arterial occlusion. It allows to investigate whether capillary rarefaction is related to a structural anatomic absence of capillaries or to a non-perfusion, reflecting both functional and structural status of the microcirculation (157). However, NVC is routinely used to evaluate structural microvascular changes without the complete estimation of the functional endothelial reserve (158). Few experiences are available on NVC in primary CVD. At present, no convincing evidence of a prognostic value of a decreased capillary density in hypertension was demonstrated (159).

### Other Techniques

In the last years, methodologies that allow functional microcirculation assessment have been used, including established methods based on medium vessels, such as flow-mediated vasodilatation (FMD) of brachial artery (160), or small digital vessels, namely peripheral arterial tonometry (PAT) (161), as well as laser doppler techniques, such as laser doppler flowmetry (LDF), laser doppler imaging (LDI) (162), laser speckle contrast imaging (LSCI), laser speckle contrast analysis (LASCA), and near-infrared spectroscopy (NIRS) (163). All of these techniques found a common basic principle: a vasodilatation in healthy arteries in response to mechanical (e.g., post-occlusive reactive hyperemia), physical (e.g., thermal challenges) and chemical stimuli (e.g., pharmacological with vasoactive substances, administered through intra-arterial infusion or iontophoresis) (138, 156). However, vascular responses are not only determined by the functional condition of the vasculature, but also by the structural status of the microvasculature. Endothelium-dependent and endothelium-independent responses can be differentiated applying exogenous NO donors (e.g., glycerol-trinitrate) or direct non-NO donors (e.g., adenosine): impaired endothelial-independent function is associated with structural vascular alterations with changes in smooth muscle cells, rather than endothelium alterations (138). All the aforementioned stimuli can be used substantially in the same way: the most frequently used are the brachial artery occlusion with a blood pressure cuff and the administration of sublingual nitroglycerin. The difference among the various techniques is the way to assess the vasodilatation. In the brachial artery FMD the respective diameter changes from the resting state of the artery are measured by ultrasound (160). PAT is a plethysmography technique that measures digital pulse volume through specific probes placed on the fingers. The average PAT amplitude (post-to-pre occlusion) of the tested arm, divided by that of the contralateral arm, is automatically calculated as the Reactive Hyperemia Index (RHI). An RHI < 1.67 is the cut off to define ED (161). The laser techniques are: LDF/LDI and LSCI/LASCA. LDF assesses the skin capillary perfusion by measuring the doppler shift induced by the scatter of the light induced by the flow of circulating red blood cells. LDI works as LDF but enables the evaluation of blood flow over a larger area of the skin compared to LDF. LSCI measures the fluctuating granular pattern produced by the reflection of the moving red blood cells illuminated by laser lights (162). LASCA is similar to LSCI where the contrast is calculated on a single pixel over a number of time frames, but has a greater temporal resolution and smaller spatial resolution than that of LSCI (164). NIRS-2D imaging provides indirect information about the microcirculation state by assessing the regional tissue oxygenation: a light in the near-infrared band penetrates the tissue and exploiting the difference between the oxygenated and deoxygenated hemoglobin in absorption spectra, estimates the balance between local arterial supply and tissue oxygen consumption. Consequently, NIRS-2D imaging provides an average value of tissue oxygen saturation (stO_2_) that is a marker of regional tissue oxygenation (163). All these techniques, especially FMD and PAT, were firstly used in the setting of atherosclerosis (55) and essential hypertension (165). Furthermore, ED, analyzed by brachial artery FMD, predicted long-term adverse CV in healthy subjects without heart disease and low clinical risk (166–168). PAT was useful in predicting non-obstructive coronary artery disease, not well predicted by the Framingham score, and late CV events in large case-series (169). FMD and PAT were confirmed to be independent predictors of CV events, with a relative risk of 0.90 per every 1% increase of FMD and 0.85 per every 0.1 increase in RHI (170). The data on the predictive values of these techniques have suggested that microvascular endothelial function assessment, which is as an earlier indicator of CV risk, could play a significant role in younger subjects or in subjects without a full-blown CVD, such as patients with autoimmune diseases. Another new technique which was recently applied in the context of autoimmune diseases is the microvascular imaging (MVI) which is a novel ultrasound modality for flow imaging, more sensitive than the conventional power doppler modality (171). It generates a high-resolution flow mapping of small vessels using adaptive image analysis to achieve an increased low-velocity blood flow stability (172). The evidence of the application of all these tools in SLE and SSc patients is reported in Table 1. In addition to the evaluation of the peripheral microcirculation of the skin, also the retinal district can be evaluated. In fact, retinal arterioles constitute another microvascular area directly and easily observed with relatively simple approaches and which share several common characteristics, including anatomic, physiological, and embryological features with heart and brain microcirculation. Recently, LDF of retinal arterioles and adaptive optics (AO), have been introduced in order to analyze small vessels morphology at the retinal field (173). Wall to lumen ratio (WLR) of retinal arterioles is the parameter which can be calculated for the evaluation of small resistance artery structure. Supporting the concept that changes in macrovasculature and microvasculature are strongly interrelated, a significant correlation among WLR values of retinal arterioles with other microvascular indexes, such as media to lumen ratio (MLR) of subcutaneous small resistance arteries, and macrovascular parameters, such as aortic and carotid stiffness, clinic and 24-h ambulatory blood pressure has been previously found in patients with hypertension (173) and initially evaluated in patients with autoimmune diseases (174).

**TABLE 1:** Applications of endothelial function assessment techniques in systemic lupus erythematosus and systemic sclerosis.

Technique	Method of vasodilatation detection after stimuli*	Finding in	
		Systemic lupus erythematosus	Systemic sclerosis
Flow-mediated vasodilatation (FMD)	Ultrasound measurement of diameter changes of the artery	– Lower FMD in patients compared to healthy subjects (176) – Lower FMD in patients carrying aPL and in patients with lupus nephritis history compared to the others (177, 178)	– Lower FMD in patients compared to healthy subjects (179) – Lower FMD in DU-patients compared to non-DU patients and FMD correlation with NVC patterns (179)
Peripheral arterial tonometry (PAT)	Measurement of digital pulse volume through specific plethysmographic finger probes	– Lower RHI in patients compared to healthy controls without correlation with SLEDAI (180)	– Lower RHI in patients compared to healthy subjects (181) – Decreased RHI values in DU-patients compared to non-DU patients and inverse correlation between RHI values and mean PAP at RHC in patients (182)
Laser doppler flowmetry/imaging (LDF/LDI)	Laser doppler assessment of the skin capillary perfusion by measuring the light scatter	– Higher microvascular dilatation in patients treated with antimalarial drugs compared to patients not in treatment (183)	– Impaired endothelium dependent vasodilatation in PAH- compared to non-PAH-patients (169)
Laser speckle contrast imaging/analysis (LCSI/LASCA)	Laser speckle contrast analysis of tissue microvascular blood perfusion	– Lower peripheral blood perfusion and impaired microvascular reactivity in patients compared to healthy subjects (184, 185) – Positive correlation of peripheral blood perfusion and number of capillaries evidenced at NVC in patients (184)	– Lower peripheral blood perfusion in patients compared to healthy subjects (181) – Lower peripheral blood perfusion in in DU- compared to non-DU patients with association of decreased skin perfusion to progression of NVC damage (186)
Near-infrared spectroscopy (NIRS)	Assessment of the regional tissue oxygenation through the near-infrared light	NA	– Lower StO_2_ values (both at baseline and at recovery time after the ischemic stimuli) in patients compared to healthy subjects (163) – Higher StO_2_ values in patients treated with sildenafil compared to patients not in treatment (163)
Microvascular imaging (MVI)	Ultrasound evaluation for flow quantification of small fingertip vessels	NA	– Peak systolic and end-diastolic flow velocities differ between patients and healthy subjects (187)

*Stimuli can be mechanical (post-occlusive reactive hyperemia), physical (thermal challenges), chemical (vasoactive drugs administered through intra-arterial infusion or iontophoresis). aPL, antiphospholipid antibodies; DU, digital ulcers; na, not applicable; NVC, nailfold video capillaroscopy; PAH, pulmonary arterial hypertension; PAP, pulmonary arterial pressure; RHC, right heart catheterization; RHI, reactive hyperemia index; SLEDAI, systemic lupus erythematosus disease activity index; StO_2_, oxygen saturation.

## Conclusion

**FIGURE 1:**
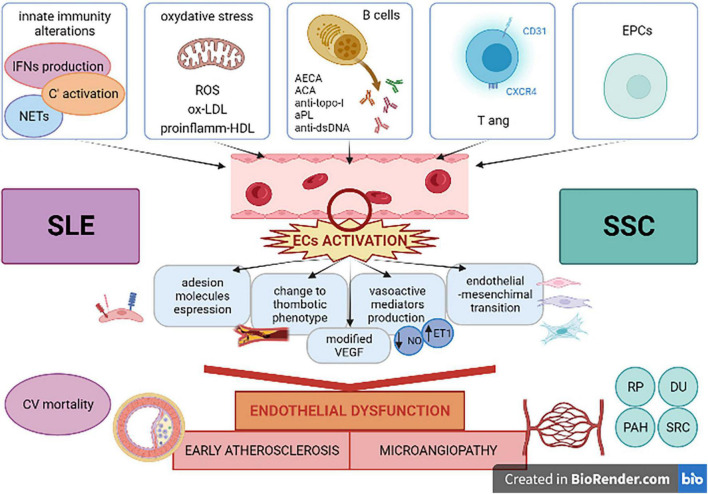
Systemic lupus erythematosus and systemic sclerosis as paradigmatic diseases in showing multiple factors involved in cardiovascular complications related to inflammatory and autoimmune processes. ACA, anti-centromere antibodies; anti-dsDNA, anti-double stranded-DNA antibodies; AECA, anti-endothelial cell antibodies; aPL, anti-phospholipid antibodies; anti-topo-I, anti-topoisomerase-I antibodies; C’, complement; CV, cardiovascular; DU, digital ulcers; ECs, endothelial cells; EPCs, endothelial progenitor cells; ET1, endothelin1; IFN, interferon; NETs, neutrophil extracellular traps; NO, nitric oxygen; ox-LDL, oxidized low-density lipoprotein; PAH, pulmonary arterial hypertension; proinflamm-HDL, proinflammatory high-density lipoprotein; ROS, reactive oxygen species; RP, Raynaud’s phenomenon; SLE, systemic lupus erythematosus; SRC, scleroderma renal crisis; SSc, systemic sclerosis; Tang, angiogenic T cells; VEGF, vascular endothelial growth factor. Created with BioRender (academic license).

Patients with systemic autoimmune diseases show an excess of CV mortality, and they represent a model for the study of pathogenetic mechanisms which have been recently evaluated as determinants in atherosclerosis and in its complications (175). In fact, the evaluation of the risk factor profile should take into account additive aspects, defined as “non-traditional drivers” which are commonly found in patients with rheumatic diseases (175). Systemic lupus erythematosus and systemic sclerosis were presented in this review as paradigmatic diseases in describing the principal factors which are involved in the determination of the excess of risk, such as ED, microangiopathy and accelerated atherosclerosis. Chronic inflammation and autoimmunity are presented as the main actors in this process and both aspects are well described in SLE and SSc (Figure 1). Despite the fact that they have many points in common, SLE represents an example of a disease in which immune system plays a central role in the organ manifestations, CV complications included, as a consequence of the state of inflammation, such a secondary condition. On the other hand, SSc is a disease in which ED is a primary dysfunction, responsible of many typical clinical features of the disease. The Framingham risk score underestimates the CV risk in patient with autoimmune diseases. Clinical tools that assess the microvasculature could represent a new approach in the CV risk evaluation, helping in the development of new models of risk prediction of our patients and changing the management of these diseases.

## Author Contributions

All authors listed have made a substantial, direct, and intellectual contribution to the work, and approved it for publication.

## Conflict of Interest

The authors declare that the research was conducted in the absence of any commercial or financial relationships that could be construed as a potential conflict of interest.

## Publisher’s Note

All claims expressed in this article are solely those of the authors and do not necessarily represent those of their affiliated organizations, or those of the publisher, the editors and the reviewers. Any product that may be evaluated in this article, or claim that may be made by its manufacturer, is not guaranteed or endorsed by the publisher.
